# Identifying myoglobin as a mediator of diabetic kidney disease: a machine learning-based cross-sectional study

**DOI:** 10.1038/s41598-022-25299-8

**Published:** 2022-12-10

**Authors:** Ruoru Wu, Zhihao Shu, Fei Zou, Shaoli Zhao, Saolai Chan, Yaxian Hu, Hong Xiang, Shuhua Chen, Li Fu, Dongsheng Cao, Hongwei Lu

**Affiliations:** 1 Health Management Center, The Third Xiangya Hospital of Central South University, Changsha, 410013 Hunan People’s Republic of China; 2 Xiangya School of Medicine, Central South University, Changsha, 410013 Hunan People’s Republic of China; 3 Department of Cardiology, The Third Xiangya Hospital, Central South University, No. 138, Tongzi, Changsha, 410013 Hunan People’s Republic of China; 4 Center for Experimental Medicine, The Third Xiangya Hospital of Central South University, Changsha, 410013 Hunan People’s Republic of China; 5 Department of Biochemistry, School of Life Sciences, Central South University, Changsha, 410013 Hunan People’s Republic of China; 6 Xiangya School of Pharmaceutical Sciences, Central South University, Changsha, 410013 People’s Republic of China

**Keywords:** Cardiology, Endocrinology, Risk factors, Diabetes complications, Type 2 diabetes, Machine learning, Biomarkers, Epidemiology

## Abstract

In view of the alarming increase in the burden of diabetes mellitus (DM) today, a rising number of patients with diabetic kidney disease (DKD) is forecasted. Current DKD predictive models often lack reliable biomarkers and perform poorly. In this regard, serum myoglobin (Mb) identified by machine learning (ML) may become a potential DKD indicator. We aimed to elucidate the significance of serum Mb in the pathogenesis of DKD. Electronic health record data from a total of 728 hospitalized patients with DM (286 DKD vs. 442 non-DKD) were used. We developed DKD ML models incorporating serum Mb and metabolic syndrome (MetS) components (insulin resistance and β-cell function, glucose, lipid) while using SHapley Additive exPlanation (SHAP) to interpret features. Restricted cubic spline (RCS) models were applied to evaluate the relationship between serum Mb and DKD. Serum Mb-mediated renal function impairment induced by MetS components was verified by causal mediation effect analysis. The area under the receiver operating characteristic curve of the DKD machine learning models incorporating serum Mb and MetS components reached 0.85. Feature importance analysis and SHAP showed that serum Mb and MetS components were important features. Further RCS models of DKD showed that the odds ratio was greater than 1 when serum Mb was > 80. Serum Mb showed a significant indirect effect in renal function impairment when using MetS components such as HOMA-IR, HGI and HDL-C/TC as a reason. Moderately elevated serum Mb is associated with the risk of DKD. Serum Mb may mediate MetS component-caused renal function impairment.

## Introduction

Diabetic kidney disease (DKD) is a common microvascular complication of diabetes^1,2^, and 50% of patients with type 2 diabetic mellitus (T2DM) will eventually develop DKD^3^. Novel biomarker discovery and accurate prediction models will aid DKD intervention and reduce disease burden^4,5^. However, currently available predictive models often lack reliable biomarkers and perform poorly^6–9^.

Components of metabolic syndrome (MetS), a cluster of risk indicators reflecting conditions such as insulin resistance (IR), dyslipidemia, and comorbidities (i.e. prothrombotic and proinflammatory states)^10^, have been widely shown to be associated with cardiovascular disease events^11,12^. MetS components are also used for DKD risk prediction, but their predictive power is not ideal. For example, based on the ADVANCE study (2012) of 18,517 T2DM patients, the COX proportional hazards regression approach indicated that baseline blood glucose was not a good predictor of DKD, whereas baseline estimated glomerular filtration rate (eGFR) and urinary albumin-creatinine ratio (ACR) dominated risk prediction^13^.

To identify any potential DKD-related indicators, we performed machine learning (ML)-based data analysis to evaluate all relevant indicators in 51,866 hospitalized patients with T2DM. Interestingly, we found serum myoglobin (Mb), a 17 kDa monomeric hemoglobin^14^ that present in human myocardial and skeletal muscle tissues to facilitate the diffusion of O_2_ from the blood to muscle mitochondria^15^, as an important feature when excluding renal function indicators (Fig. S1). Since this is only a preliminary analysis finding and the influences of missing values and related diseases are not considered, further in-depth analysis is needed. From a molecular mechanism perspective, subclinical elevations in serum Mb have recently been observed in DM and chronic kidney disease (CKD)^16^, which may be explained by diabetic muscle damage and abnormal glucose and lipid metabolism, suggesting that MetS components may be contributing to elevated serum Mb levels^17–20^. Further, tissue damage caused by Mb has been confirmed at the molecular level. For example, studies have shown that Mb interacts with glucose to form end-of-glycosylation end products (AGEs), which ultimately lead to intramolecular and/or intermolecular cross-linking of long-lived proteins, causing chronic inflammation^21–23^. However, the importance of serum Mb in DKD has not been evaluated, nor has serum Mb been investigated as a risk factor in models of DKD. Moreover, the pathological role of serum Mb as a mediator in MetS component-induced renal function impairment has not been reported.

Therefore, to clarify the possible pathophysiological significance of serum Mb in DKD, we used a cross-sectional study design and obtained a total of 51,866 electronic health records (EHRs) of hospitalized patients with T2DM in the Third Xiangya Hospital of Central South University. We established DKD ML models incorporating serum Mb and simultaneously evaluated the models’ performance and feature importance to verify the importance of serum Mb in DKD. Logistic regression-based restricted cubic spline (RCS) models were applied to further assess the relationship between serum Mb and DKD. We explored the causal relationship between MetS components, serum Mb, and DKD using causal mediation analysis, aiming to test the hypothesis that serum Mb mediates the induction of renal function impairment by MetS components.

## Materials and methods

### Clinical data sources

After obtaining the data access authorization from the Information Network Center of the Third Xiangya Hospital, we retrospectively collected the EHRs of 51,866 hospitalized patients from the Hospital Information System (HIS). The Third Xiangya Hospital of Central South University is located in Changsha City, Hunan Province. It is a grade A tertiary general hospital serving the middle reaches of the Yangtze River. This study was approved by the IRB of the Third Xiangya Hospital of Central South University (No: 21112). All data had been completely anonymized prior to our access to the HIS. Because the patients' personally identifiable information had been effectively protected, the IRB of the Third Xiangya Hospital of Central South University waived the requirement for informed consent. The present study was conducted in accordance with the guidelines of the Declaration of Helsinki.

### Study population

**Figure 1:**
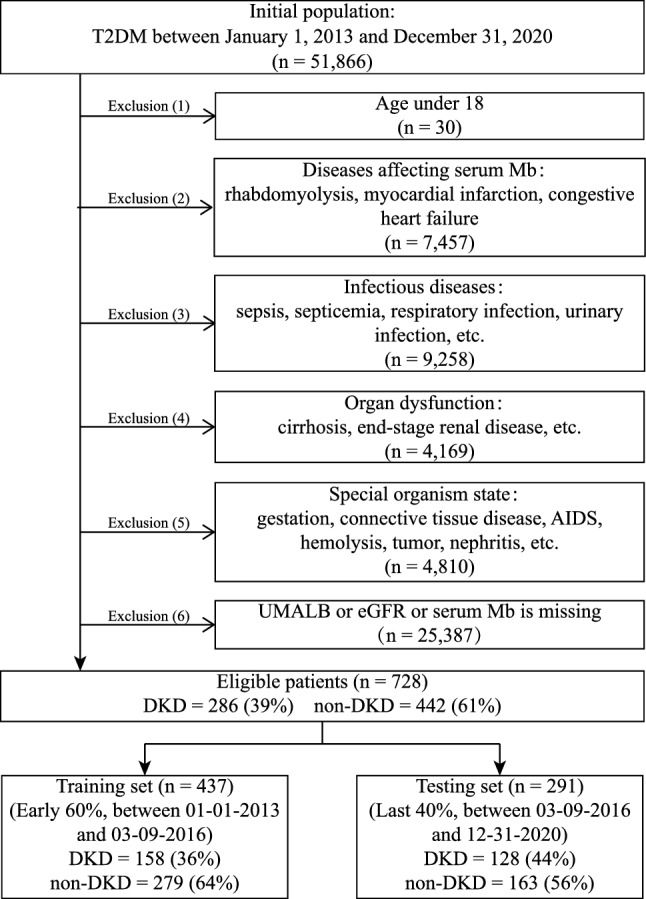
Inclusion and exclusion criteria of study population. Electronic health records (EHRs) of 51,866 hospitalized patients with type 2 diabetic mellitus (T2DM) were collected. Patients meeting the following exclusion criteria were removed: (1) under 18 years of age (n = 30), (2) diseases affecting serum myoglobin (Mb) (n = 7457), (3) infectious diseases (n = 9258), (4) organ dysfunction (n = 4169), (5) special body status (n = 4810), and (6) missing urine microalbumin (UMALB), estimated glomerular filtration rate (eGFR) and/or serum Mb data (n = 25,387). Finally, 728 patients were eligible for our study. The training set and testing set sample sizes were 437 and 291, respectively.

The EHRs of 51,866 hospitalized patients with T2DM in the Third Xiangya Hospital from January 1, 2013 to December 31, 2020 were collected. Exclusion criteria: (1) under 18 years of age (n = 30), (2) diseases affecting Mb (n = 7457), (3) infectious diseases (n = 9258), (4) organ dysfunction (n = 4169), (5) special body status (n = 4810), and (6) missing urine microalbumin (UMALB), eGFR and/or serum Mb data (n = 25,387). Finally, 728 patients (DKD = 286, non-DKD = 442) were eligible for the study. Eligible patients were divided into training set (60%, earlier than March 9, 2016) and testing set (40%, later than March 9, 2016), including 437 (DKD = 158, non-DKD = 279) and 291 patients (DKD = 128, non-DKD = 163), respectively, based on hospitalization date. The inclusion and exclusion criteria of the study population are presented in Fig. 1, and the demographic and clinical features are available in Table 1.

**Table 1:** Demographic and clinical features.

Feature	Non-DKD (n = 442)	DKD (n = 286)	P
Male	232 (52.5)	161 (56.3)	0.352
Age	60.00 [52.00, 68.75]	63.00 [54.00, 70.00]	0.013
BMI (kg/m^2^)	23.74 [22.11, 25.95]	24.22 [22.04, 26.14]	0.577
mean SBP (mmHg)	130.29 [120.19, 139.00]	139.57 [129.91, 148.86]	< 0.001
mean DBP (mmHg)	77.34 [72.16, 83.00]	78.38 [74.00, 83.78]	0.040
mean HR (beats/minute)	77.00 [74.63, 79.25]	77.07 [74.43, 80.86]	0.204
Hypertension	217 (49.1)	208 (72.7)	< 0.001
Hyperlipidemia	132 (29.9)	83 (29.0)	0.873
Diabetic retinopathy	123 (27.8)	165 (57.7)	< 0.001
Diabetic neuropathy	231 (52.3)	207 (72.4)	< 0.001
Coronary heart disease	64 (14.5)	56 (19.6)	0.087
ACCI	5.00 [4.00, 7.00]	6.00 [5.00, 8.00]	< 0.001
FRS	14.00 [11.00, 16.00]	15.00 [12.00, 17.00]	< 0.001
UMALB (mg/L)	14.60 [8.35, 28.00]	203.35 [41.64, 721.58]	< 0.001
Scr (μmol/L)	68.00 [58.00, 80.00]	96.50 [72.00, 141.25]	< 0.001
eGFR (ml/min/1.73m^2^)	96.26 [78.26, 115.96]	66.76 [41.16, 92.65]	< 0.001
BUN (mmol/L)	5.39 [4.48, 6.44]	6.56 [5.27, 8.53]	< 0.001
Mb (ng/mL)	48.65 [37.70, 65.52]	80.47 [56.92, 120.67]	< 0.001
CK (U/L)	79.00 [57.00, 111.00]	89.00 [62.00, 132.00]	0.014
CK-MB (U/L)	16.00 [13.00, 20.38]	18.00 [13.00, 22.00]	0.084
LDH (U/L)	195.00 [171.00, 225.00]	203.00 [177.00, 247.00]	0.013
**20 MetS components**			
Fasting C-peptide (ng/mL)	1.48 [0.90, 2.12]	1.71 [1.10, 2.46]	< 0.001
120 min C-peptide (ng/mL)	2.49 [1.39, 4.43]	2.72 [1.58, 4.20]	0.316
HOMA-IR	1.87 [0.98, 3.21]	1.96 [1.08, 3.48]	0.228
Gutt index	133.69 [119.65, 160.31]	133.05 [120.73, 155.43]	0.811
TyG index	6.91 [6.38, 7.50]	7.03 [6.45, 7.50]	0.412
I/G 0 min	0.15 [0.10, 0.25]	0.18 [0.12, 0.30]	0.002
I/G 120 min	0.20 [0.12, 0.37]	0.22 [0.14, 0.37]	0.472
IGI	0.25 [0.09, 0.62]	0.24 [0.11, 0.58]	0.700
IGI/HOMA-IR	0.16 [0.04, 0.42]	0.15 [0.04, 0.44]	0.982
HOMA-BETA	21.03 [9.66, 46.36]	25.87 [12.59, 49.02]	0.044
Fasting glucose (mmol/L)	8.14 [6.30, 10.75]	8.00 [6.12, 10.38]	0.272
120 min glucose (mmol/L)	11.60 [8.70, 14.10]	11.40 [8.53, 14.64]	0.898
GHBA1C (%)	8.55 [7.00, 10.20]	8.10 [6.80, 9.70]	0.033
HGI	0.25 [− 0.91, 1.79]	− 0.01 [− 1.06, 1.33]	0.022
TC (mmol/L)	4.45 [3.84, 5.29]	4.49 [3.80, 5.41]	0.867
TG (mmol/L)	1.58 [1.03, 2.10]	1.62 [1.16, 2.20]	0.133
LDL-C (mmol/L)	2.30 [1.85, 2.82]	2.22 [1.90, 2.74]	0.668
HDL-C (mmol/L)	1.15 [0.98, 1.34]	1.10 [0.99, 1.30]	0.343
HDL-C/TC (%)	0.25 [0.21, 0.31]	0.24 [0.21, 0.29]	0.430
TG/HDL ratio (%)	1.35 [0.86, 2.17]	1.47 [0.93, 2.15]	0.326

Data are presented as median [interquartile range] for continuous variables or number (%) for categorical variables. P-values for differences between groups were based on the Wilcoxon test for continuous variables and the chi-square test for categorical variables. Details of features calculation are shown in Table S1. *Non-DKD* Diabetes without any kidney disease, *SBP* systolic blood pressure, *DPB* diastolic blood pressure, *HR* heart rate, *ACCI* age-adjusted Charlson Comorbidity Index, *FRS* Framingham Risk Score, *UMALB* urinary albumin excretion, *eGFR* estimated glomerular filtration rate, *Scr* serum creatinine, *BUN* urea, Mb myoglobin, *CK* creatine kinase, *CK-MB* creatine kinase Isoenzyme, *LDH* lactate dehydrogenase, *HOMA*-*IR* Homeostatic Model Assessment of Insulin Resistance, *TyG index* triglyceride-glucose index, *I/G 0 min* fasting Insulin to glucose ratio, *I/G 120 min* 120 min Insulin to glucose ratio, *IGI* insulinogenic index, *IGI/HOMA-IR* IGI to HOMA-IR ratio, *HOMA-BETA* Homeostasis Model of Assessment for beta-cell function, *GHBA1C* glycated hemoglobin A1c, *HGI* hemoglobin glycation index, *TC* total cholesterol, *TG* triglycerides, *LDL-C* low-density lipoprotein cholesterol, *HDL-C* high-density lipoprotein cholesterol and *TG/HDL ratio* triacylglycerol to high density lipoprotein cholesterol ratio.

### Data preparation

From the EHRs of T2DM, we extracted a total of 157 features in the following 7 domains: demographic information, hospitalization-related information, general information, laboratory tests, diagnosis, medication, and others. The data domain categories and descriptions are listed in Table 2. The details of feature calculation are shown in Table S1.

**Table 2:** Data domain categories and descriptions.

Domain	Descriptions	Data type	Number of features
Demographics	Gender, age	Binary, numeric	2
Hospitalization characteristics	Hospitalization date, leave date, hospital day, admission department, discharge department	Date, numeric, integer	5
General information	Height, weight, BMI, mean SBP, SD SBP, max SBP, min SBP, mean DBP, SD DBP, max DBP, min DBP, mean HR, SD HR, max HR, min HR	Numeric	15
Laboratory tests	GHBA1C, fasting glucose, fasting insulin, fasting C-peptide, 120 min glucose , 120 min insulin, 120 min C-peptide, TC, TG, LDL-C, HDL-C, HDL-C/TC ratio, HGI, TyG index, TG/HDL ratio, HOMA-IR, HOMA-BETA, IGI, IGI/HOMA-IR, I/G 0 min, I/G 120 min, Gutt index, WBC, Hb, PLT, MCV, MCH, MCHC, Neu, Lym, Lym%, Neu%, CK, LDH, CK-MB, Mb, troponin, BNP, UMALB, BUN, Scr, UA, eGFR, C-reactive protein, hypersensitive C-reactive protein, NLR, PLR	Numeric, integer	47
Diagnoses	T2DM, hyperlipidemia, DKD, CKD, stages of CKD, renal anemia, renal bone disease, DR, stages of DR, DN, diabetic foot, CAD, heart failure, stages of heart failure, extremity arteriosclerosis, carotid atherosclerosis, renal artery stenosis and sclerosis, cerebral arteriosclerosis, retinal arteriosclerosis, acute MI, old MI, congestive heart failure, stroke, end-stage renal disease, sepsis, septicemia, HT, stages of HT, peptic ulcer, fatty liver disease, cirrhosis, viral hepatitis, chronic bronchitis, COPD, asthma, arrhythmia, atrial flutter and atrial fibrillation, pregnancy period, hyperthyroidism, hypothyroidism, hyperparathyroidism, hashimoto's thyroiditis, brain tumors, breast tumors, lung tumors, digestive system tumors, urinary system tumors, thyroid tumors, tumors of the male reproductive system, tumors of the female reproductive system, leukemia, lymphoma, metastasis, respiratory system infection, urinary system infection, rheumatoid or connective tissue disease, other cerebrovascular diseases or TIA, other liver diseases, HIV/AIDS, hemiplegia, Alzheimer’s disease, rhabdomyolysis, hemolysis, nephritis, infection, tumors	Numeric, binary	66
Medications	Diuretics, beta-blockers, calcium-channel blockers, ACE inhibitor, angiotensin-receptor blocker, vasodilators, statins, fibrates, other lipid-lowering agents, sulfonylureas, glinides, biguanides, thiazolidinediones, alpha-glucosidase inhibitors, DPP-4 inhibitor, SGLT-2 inhibitor, insulin, antihypertensive agents, lipid-lowering agents, hypoglycemic agents	Binary	20
Others	ACCI, FRS	Integer	2

*SBP* systolic blood pressure, *DPB* diastolic blood pressure, *HR* heart rate, *GHBA1C* glycated hemoglobin A1c, *TC* total cholesterol, *TG* triglycerides, *LDL-C* low-density lipoprotein cholesterol, *HDL-C* high-density lipoprotein cholesterol, *WBC* white blood cell count, *Hb* hemoglobin, *PLT* platelet count, *MCV* mean corpuscular volume, *MCH* mean corpuscular hemoglobin, *MCHC* mean corpuscular hemoglobin concentration, *Neu* neutrophils, *Lym* lymphocyte, *Neu*% percentage of Neu, *Lym*% percentage of Lym, *CK* creatine kinase, *LDH* lactate dehydrogenase, *CK-MB* creatine kinase Isoenzyme, *Mb* myoglobin, *UMALB* urinary albumin excretion, *BUN* urea, *Scr* serum creatinine, *UA* uric acid, *HGI* hemoglobin glycation index, *TyG index* triglyceride-glucose index, *TG/HDL ratio* triacylglycerol to high density lipoprotein cholesterol ratio, *HOMA-IR* homeostatic model assessment of insulin resistance, *IGI* insulinogenic index, *IGI/HOMA-IR* IGI to HOMA-IR ratio, *HOMA-BETA* homeostasis model of assessment for beta-cell function, *I/G 0 min* fasting insulin to glucose ratio, *I/G 120 min* 120 min insulin to glucose ratio, *eGFR* estimated glomerular filtration rate, *NLR* neutrophil-to-lymphocyte ratio, *PLR* platelet-to-lymphocyte ratio, *CKD* chronic kidney disease, *DR* diabetic retinopathy, *DN* diabetic neuropathy, *CAD* coronary artery disease, *MI* myocardial infarction, *HT* hypertension, *ACCI* age-adjusted Charlson comorbidity index, *FRS* Framingham risk score.

Diagnoses were organized using the ICD -9 and ICD-10 hierarchies. These diseases were diagnosed by experienced doctors who were licensed to practice medicine. T2DM was diagnosed according to the World Health Organization (WHO) criteria (1999)^24^. DKD was diagnosed by the NKF/KDOQI (2007) classification^25^. Serum Mb was tested by Mb-LATEX CN from Denka Seiken (Reference range 0–70 ng/mL).

The components of MetS have the following three sets of features: IR and β-cell function were evaluated by fasting C-peptide, 120 min C-peptide, Homeostatic Model Assessment of Insulin Resistance^26^ (HOMA-IR), Gutt index^27^, triglyceride-glucose (TyG) index^28^, fasting insulin to glucose ratio^29^ (I/G 0 min), 120 min insulin to glucose ratio^29^ (I/G 120 min), insulinogenic index^30^ (IGI), IGI to HOMA-IR ratio^31^ (IGI/HOMA-IR), and Homeostasis Model of Assessment for beta-cell function^32^ (HOMA-BETA). Glucose metabolism indicators included fasting glucose, 120 min glucose, glycated hemoglobin A1c (GHBA1C), and hemoglobin glycation index^33^ (HGI). Lipid metabolism was assessed by total cholesterol (TC), triglycerides (TG), low-density lipoprotein cholesterol (LDL-C), high-density lipoprotein cholesterol (HDL-C), HDL-C/TC, and TG/HDL-C ratio^34^.

### ML algorithms

Two popular ML methods, extreme gradient boosting (XGBoost) and random forest (RF), were used to build DKD classification models based on EHR data. RF, first proposed by Breiman in 2001^35^, is an ensemble of unpruned classification or regression trees created using bootstrap samples of training data and random feature selection in tree induction. XGBoost is an efficient and scalable implementation of the gradient boosting framework^36^. It is used to develop models in a sequential stagewise fashion like other boosting methods and generalize them by allowing optimization of arbitrary differentiable loss functions. RF and XGBoost were applied using the randomForest (4.6–14)^37^ and xgboost (1.3.2.1)^38^ R packages, respectively. All ML methods used grid search and fivefold cross-validation for parameter adjustment. The final hyperparameters for the DKD models are listed in Table S2.

### Model validation

The evaluation indicators of the confusion matrix, including sensitivity (SE), specificity (SP), positive predictive value (PPV), negative predictive value (NPV), recall, F1, and balanced accuracy (BA), were used to analyze the discrimination ability of DKD ML models. The probability scores output by the models were used to calculate the area under the receiver operating characteristic (ROC) curve (AUC). These statistical parameters are defined as follows:

$$SE=\frac{\mathrm{TP}}{TP+FN},$$

$$SP=\frac{\mathrm{TN}}{TN+FP},$$

$$\mathrm{PPV}=\frac{\mathrm{T}P}{TP+FP},$$

$$\mathrm{NPV}=\frac{\mathrm{T}N}{TN+FN},$$

$$\mathrm{Recall}=\frac{\mathrm{T}P}{TP+FN},$$

$$\mathrm{F}1=\frac{2\times PPV\times \mathrm{recall}}{PPV+recall},$$

$$BA=\frac{\mathrm{SE}+\mathrm{SP}}{2},$$

where true positive (TP) is the number of correctly classified objects, true negative (TN) is the number of correctly misclassified objects, false positive (FP) is the number of incorrectly classified objects, and false negative (FN) is the number of incorrectly misclassified objects.

### Feature evaluation and model interpretation

Feature importance of RF was estimated by (1) mean decrease in prediction accuracy without the feature in the model and (2) mean decrease in the Gini index, a measure of impurity of the dataset (i.e. risk of misclassifying data), by including the feature. For both parameters, the higher the score, the more important the feature.

We evaluated feature contributions to model predictions using an interpretability method called SHapley Additive exPlanation (SHAP). SHAP is based on the Shapley value, a concept introduced in the 1950s to measure each player’s contribution to a collaborative game^39^. In 2017, Lundberg and Lee proposed a broadly applicable SHAP method to interpret a variety of models, including regression and classification^40,41^. For binary classification in XGBoost, the output of the model is the log odds ratio, that is, the SHAP values sum up to the log loss of the model for each sample. The function is defined as follows

$$z={\varphi }_{0}+\sum_{i=1}^{M}{\varphi }_{i}{x}_{i}^{^{\prime}},$$

where $z$ is the log odds ratio, ${x}^{^{\prime}}$ is a simplified binary feature vector containing values 1 or 0 for observed and missing features, M is the number of simplified input features, and ${\varphi }_{i}$ is the attribution value of the ith molecular descriptor or the SHAP value. In probability theory, $z$ is the corresponding value of probability p after a certain transformation, and the transformation function is defined as follows

$$z=\mathrm{log}\frac{p}{1-p},$$

where $p$ varies monotonically in the range (0, 1) and $z$ varies from infinitesimal to infinity. A feature is predicted to be a risk factor if its $z$-value is greater than 0. The larger the z-value, the more likely it is a risk factor rather than a protective factor. Therefore, the SHAP method can help us understand the model more intuitively. The SHAPforxgboost (0.1.1) and fastshap (0.0.5) R packages were applied in the analysis.

### Decision curve analysis (DCA)

We used DCA to assess the clinical utility of ML models. A decision curve is generated by plotting net benefit against a range of threshold probabilities. Decision curves include benefits and harms on the same scale, so they can be directly compared, supporting comparisons between models. The net benefit is calculated as

$$Net benefit=\frac{True positive}{N}-\frac{True negtive}{N}\times \frac{ {P}_{t}}{1-{P}_{t}},$$

where N is the total sample size and ${P}_{t}$ represents the threshold probability. Two extreme strategies, classifying for all and classifying for none, were added for reference. The R package rmda (1.6) was used.

### RCS models

The association between serum Mb and DKD was evaluated on a continuous scale with RCS curves based on logistic regression models. To balance best fit and overfitting, the number of knots was chosen empirically to be 4. The serum Mb associated with the lowest risk of DKD was the concentration with the lowest odds ratio on the spline curve. Odds ratios were adjusted for gender, age, BMI, hyperlipidemia, hypertension, age-adjusted Charlson comorbidity index (ACCI), and hospitalization date.

### Causal mediation analysis

Causal mediation analysis identifies potential pathways to explain the observed association between a given exposure and a particular outcome^42^, examining how a third intermediate variable, the mediator, is related to the observed outcome relationship. By using a counterfactual framework, causal mediation analysis allows us to decompose the total effect (TE) into direct effects (DE) and indirect effects (IE). In our hypothetical model, we defined TE as the effect of MetS components on renal function impairment, DE as the effect of MetS components on renal function impairment after removing the individual effect of Mb on renal function impairment, and IE as the effect of MetS components on renal function impairment specially associated with Mb. A directed acyclic graph (DAG) illustrating mediation process is shown in Fig. 5A. Through the causal mediation analysis, we estimated the mediation effect of serum Mb based on logistic regression in all eligible patients, DKD or non-DKD subgroups. According to previous DKD investigators^43,44^, we adjusted and standardized the MetS components, Mb, UMALB, and eGFR for 7 variables: gender, age, BMI, hyperlipidemia, hypertension, ACCI, and hospitalization date. The proportion of serum Mb-mediated renal function impairment due to the MetS components was calculated as

$$Prop.Mediated\left(average\right)=\frac{\mathrm{log}\left(IE\right)}{\mathrm{log}\left(IE\right)+\mathrm{log}\left(DE\right)},$$

Causal mediation analysis was performed using the R package mediation (4.5.0).

### Statistical analysis

R software (version 4.0.3) was used as a statistical analysis tool in this study. Data are presented as median [interquartile range] for continuous variables or number (%) for categorical variables. Differences between groups were based on the Wilcoxon test for continuous variables and the chi-square test for categorical variables. Relationships between MetS components, serum Mb, and DKD were assessed with the Spearman’s correlation coefficient (r_s_), controlling for 7 variables: gender, age, BMI, hyperlipidemia, hypertension, ACCI, and admission date. All statistical significance was defined as P < 0.1. Missingness was addressed by setting missing categorical data to 0, whereas for numerical data, imputation was completed using the predictive mean matching (PMM) strategy.

### Bias

Observer bias may arise from the data on patients hospitalized in a grade A tertiary general hospital, as these patients may have more severe than average DM or DKD. Besides, selection bias caused by exclusion of UMALB missing individuals brings DKD rate elevation but comparable cardiovascular risks, leading to limited representation (Table S3). However, the significance and possible mediator role of serum Mb in DKD, as a common rule, was mainly focused in our study, and we believe that the bias did not greatly affect our conclusion.

## Results

### Participant characteristics

The clinical characteristics of the participants are presented in Table 1. There were no significant between-group differences in age, sex, and BMI. However, the DKD group showed significantly more diabetes complications such as diabetic retinopathy and diabetic neuropathy. Importantly, serum Mb was significantly elevated in the DKD group, consistent with previous studies^45^.

### DKD model performance

To verify and evaluate the significance of serum Mb in DKD, we built DKD ML models incorporating serum Mb, taking advantage of its freedom from collinearity in processing high-dimensional EHR data on disease phenotypes and clinical outcomes^46–48^, aiming to discover important features overlooked by traditional methods. We first accessed the model performance to ensure that the following feature importance analysis and model interpretation are accurate and reliable.

**Figure 2:**
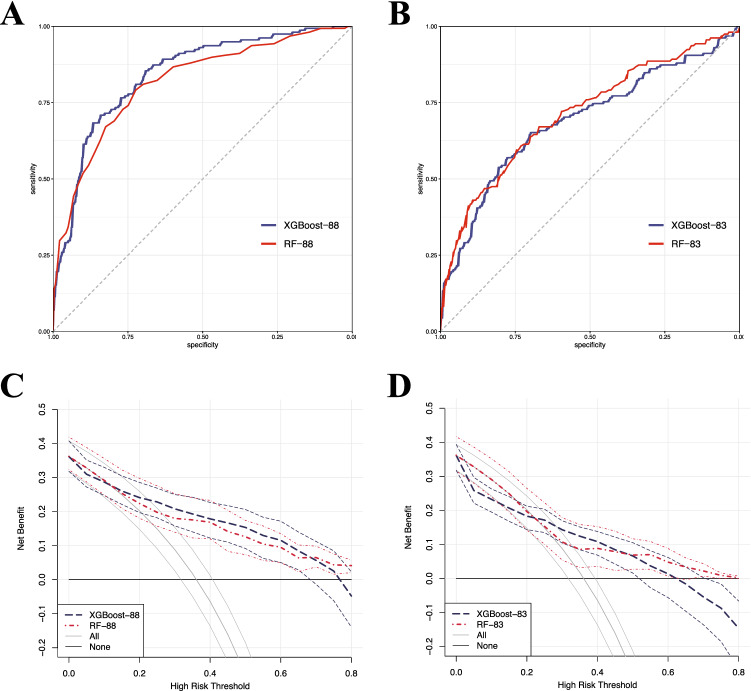
Receiver operating characteristic (ROC) curves and decision curve analysis (DCA) results in the testing set. (**A,B**) ROC curves of XGBoost and RF models based on 88 features and 83 features, respectively. (**C,D**) DCA for classifying DKD in T2DM patients by machine learning (ML) models based on 88 features and 83 features, respectively. The net benefit and 95% confidence interval for the ML models are shown with blue and red dashed lines. Two extreme strategies, classifying for all and classifying for none, are represented by solid grey and solid black lines.

After removing features with a missing ratio of more than 40% or zero variance, a total of 88 important features (including renal function) were available for DKD modeling to obtain high-accuracy DKD classification models. Two popular ML algorithms, XGBoost and RF algorithms, have been applied, named XGBoost-88 and RF-88, respectively. The model performances in the testing set are summarized in Table 3 and the ROC curves are shown in Fig. 2A. Overall, both approaches performed satisfactorily (e.g. all AUCs > 0.85). Clearly, the best classifier based on the RF algorithm was able to achieve AUC values of 0.884, BA values of 0.798, and PPV values of 0.854 in the testing set.

**Table 3:** Performance of machine learning algorithms in testing set (n = 291).

Models	SE	SP	PPV	NPV	Recall	F1	BA	AUC
XGBoost-88	0.719	0.853	0.793	0.794	0.719	0.754	0.786	0.864
RF-88	0.688	0.908	0.854	0.787	0.688	0.762	0.798	0.884
XGBoost-83	0.523	0.865	0.753	0.698	0.523	0.618	0.694	0.806
RF-83	0.477	0.920	0.824	0.691	0.477	0.604	0.698	0.824

We also applied XGBoost and RF algorithms to renal function exclusion dataset (contained 83 features and removed UMALB, serum creatinine (Scr), eGFR, blood urea nitrogen (BUN), and serum uric acid (SUA)), aiming to find the features hidden by renal function features. XGBoost-83 and RF-83 were named respectively. Model performances in the testing set are summarized in Table 3 and ROC curves are shown in Fig. 2B. On the whole, the performances of both approaches were relatively satisfactory (e.g. all AUCs > 0.80). Compared with the model including renal function features, BA decreased by about 0.1, SP did not change much, and SE decreased by 0.2 in the renal-function-excluded-model, indicating that renal function indicators are crucial for identifying DKD, while other features are important for ruling out DKD.*SE* sensitivity, *SP* specificity, *PPV* positive predictive value, *NPV* negative predictive value, *BA* balanced accuracy, *AUC* area under curve.

### DCA for ML models

To assess the clinical utility of several ML models we built, DCA was applied to these ML models (Fig. 2C,D). For both XGBoost/RF-88 and XGBoost/RF-83, the net benefits of two models were similar across the range of threshold probabilities. When the threshold probability was between 0.4 and 0.6, both models outperformed the reference strategies. These results of DCA show the consistency and efficiency of our models and are consistent with our model performance data (Table 3).

### Feature importance analysis and model interpretation

Next, to identify important features hidden from indicators of renal function and attest the importance of serum Mb for DKD, feature importance analysis and model interpretation were applied, the reliability of which has been proved by model performance and DCA.

**Figure 3:**
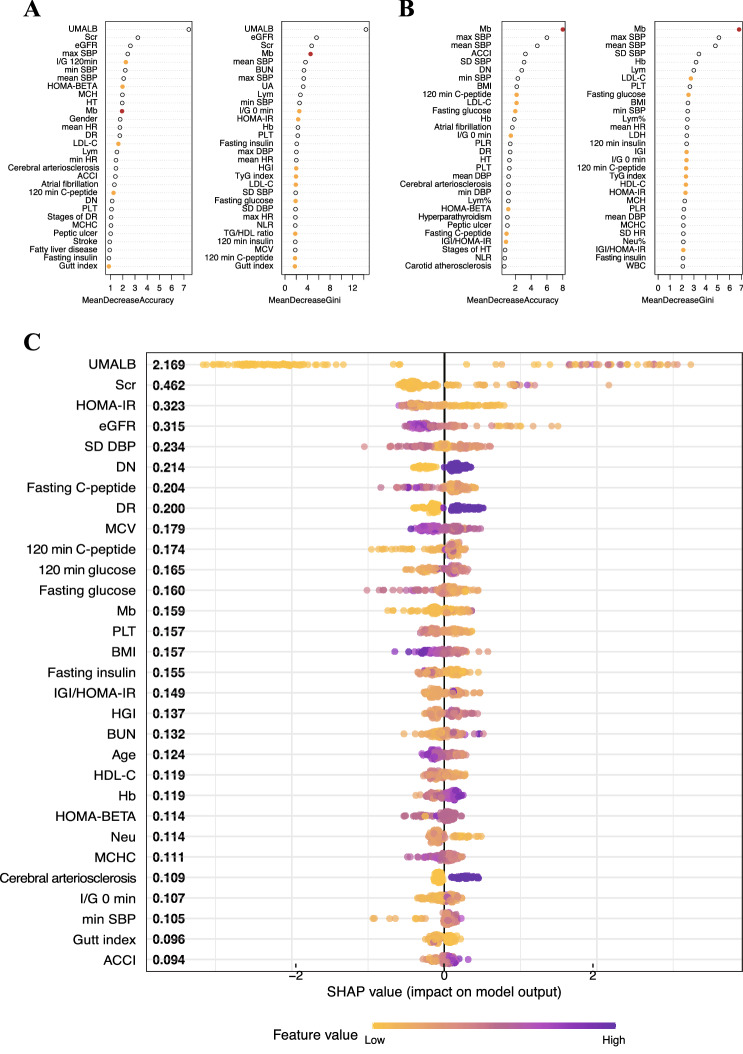
Feature importance analysis and model interpretation results. (**A,B**) Feature importance was estimated by mean decrease accuracy (left) and mean decreased Gini index (right) based on RF-88 and RF-83, respectively. Serum Mb and metabolic syndrome (MetS) components are indicated by red and orange dots, respectively. (**C**) Global and local importance for top 30 important features detecting by SHapley Additive exPlanation (SHAP).

The top 30 important variables for RF-88 and RF-83 are presented in Fig. 3A,B, and two estimators, mean decrease accuracy and mean decrease Gini, have been applied to measure feature importance. In RF-88, UMALB, eGFR, and Scr were listed as top-ranked features. It’s worth noting that serum Mb consistently ranked in the front. Meanwhile, MetS components such as I/G 120 min, I/G 0 min, HOMA-BETA, HOMA-IR, TyG index, Gutt index, 120 min C-peptide (reflecting IR and β-cell function), HGI, fasting glucose (reflecting glucose metabolism), and LDL-C, TG/HDL ratio (reflecting lipid metabolism) were on the list. Intriguingly, in RF-83, serum Mb emerged as the most important feature under all estimators. Meanwhile, MetS components such as 120 min C-peptide, fasting C-peptide, IGI, IGI/HOMA-IR, HOMA-BETA, HOMA-IR, I/G 0 min, TyG index (reflecting IR and β-cell function), fasting glucose (reflecting glucose metabolism), LDL-C, HDL-C (reflecting lipid metabolism) were relatively important features.

To overcome the black-box nature of XGBoost and RF algorithms and better understand the prediction models, model interpretation based on XGBoost-88 correct classifications was carried out using the SHAP approach. The top 30 important features identified by SHAP are shown in Fig. 3C. We found that the renal function features UMALB, Scr, and eGFR remained dominant. Serum Mb (ranked 13th) was recognized as an important feature. MetS components, including HOMA-IR, fasting C-peptide, 120 min C-peptide, IGI/HOMA-IR, HOMA-BETA, I/G 0 min, Gutt index (reflecting IR and β-cell function), 120 min glucose, fasting glucose, HGI (reflecting glucose metabolism), and HDL-C (reflecting lipid metabolism) were important features. These results are consistent with the feature importance analysis described above. We noted that higher serum Mb, worsening renal functions (i.e. higher UMALB and Scr and lower eGFR), more diabetes comorbidities (i.e. diabetic neuropathy and diabetic retinopathy), worsening IR and β-cell function (higher IGI/HOMA-IR, AC, HOMA-BETA, and I/G 0 min), and worse glucose control (e.g. higher HGI and AGLU) were associated with DKD.

Our results suggest that in addition to renal function features, serum Mb is a hidden important feature in DKD, and that elevated serum Mb is associated with DKD classification. Meanwhile, MetS components also play a key role in helping to build ML decision-making algorithms in DKD.

### RCS identifies the association between serum Mb and DKD

**Figure 4:**
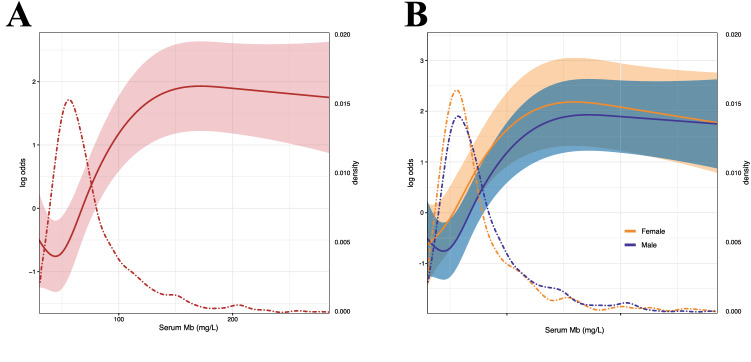
Restricted cubic spline models for log odds ratios of DKD versus serum Mb on a continuous scale. Analyses were adjusted for gender, age, BMI, hyperlipidemia, hypertension, ACCI, and hospitalization date. (**A**) The red solid line is the multivariable adjusted log odds ratio, and the shaded area shows the 95% confidence interval derived from restricted cubic spline regressions with four knots. The red dashed curve represents the distribution density of the population with different serum Mb levels. (**B**) The orange and blue solid lines are the multivariable adjusted log odds ratio for female and male subgroups, respectively, and the shaded areas show the 95% confidence intervals derived from restricted cubic spline regressions with four knots. The orange and blue dashed curves show the distribution density of populations with different serum Mb levels in female and male subgroups, respectively.

By using ML algorithm-based variable importance analysis and SHAP, we verified the importance of serum Mb for DKD discrimination, supporting serum Mb as a potential important feature of DKD. Next, we further evaluated the association between serum Mb and DKD using RCS on a continuous scale (Fig. 4A,B). We found that the odds ratio was greater than 1 when serum Mb was greater than about 80, indicating that moderately elevated serum Mb is associated with DKD risk. Similar results were seen in male and female patients. However, when adjusting for eGFR, serum Mb was not significant associated with DKD across the range (Fig S2), indicating a strong correlation between serum Mb and eGFR.

### Causal mediation analysis reflects serum Mb as a possible mediator of MetS component-induced renal function impairment

**Figure 5:**
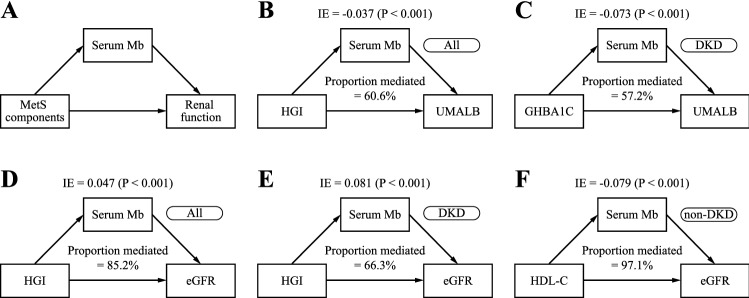
Serum Mb mediates the relationship between MetS components and renal function. (**A**) A directed acyclic graph (DAG) illustrating the mediation process. (**B–F**) Results of causal mediation analysis of 5 significant indirect effects (IE) for serum Mb in all eligible patients, DKD or non-DKD subgroups. (**B,C**) significant IE seen in all eligible patients and DKD subgroup using UMALB as an indicator of renal function. (**D,E,F**) Significant IE seen in all eligible patients, DKD and non-DKD subgroups, respectively, using eGFR as an indicator of renal function.

Based on the aforementioned ML variable importance analysis, SHAP approach, and RCS results, we noticed that serum Mb and MetS components were closely related to DKD. However, studies have shown that serum Mb is elevated in the case of metabolism disorders, and Mb may directly or indirectly cause tissue damage. Thus, we systematically evaluated whether serum Mb mediates renal impairment associated with the MetS components (20 indicators), and the most significant results are shown in Fig. 5. The spearman correlation coefficient between MetS components and among MetS components, serum Mb, and renal function indicators are shown in Fig. S3 and Table S4, indicating that each component was irreplaceable. Renal function impairment (or DKD severity) was evaluated by eGFR and UMALB.

Significant IE and TE were found in 8 out of 20 causal mediation models when evaluating renal impairment using eGFR across all observations. Six indicators (HGI, 120 min glucose, fasting glucose, HOMA-IR, IGI/HOMA-IR and IGI in descending order of proportion mediated, the same below) reflecting IR, β-cell function, and glucose metabolism in the DKD subgroup and two indicators (HDL-C, HDL-C/TC) reflecting lipid metabolism in the non-DKD subgroup showed significant IE and TE (see Table 4 for significant results and Table S5 for full results).

**Table 4:** DE, IE, and TE of MetS components on renal function impairment (eGFR) mediated through serum Mb (Significant).

Group	MetS components	Number of patients (%)	P_IE_	IE [95% CI]	DE [95% CI]	TE [95% CI]	Proportion medited (%)
All	Fasting C-peptide	728 (100.0)	0.02	[− 0.07, − 0.01]	[− 0.30, − 0.16]	[− 0.35, − 0.20]	13.5
	HOMA-IR		< 0.001	[0.02, 0.08]	[0.09, 0.22]	[0.15, 0.28]	22.1
	IGI		< 0.001	[− 0.10, − 0.03]	[− 0.32, − 0.19]	[− 0.39, − 0.26]	17.6
	IGI/HOMA-IR		0.02	[− 0.07, 0.00]	[− 0.17, − -0.04]	[− 0.21, − 0.08]	20.6
	Fasting glucose		< 0.001	[0.02, 0.09]	[0.10, 0.21]	[0.15, 0.28]	23.9
	120 min glucose		0.04	[0.00, 0.07]	[0.00, 0.11]	[0.03, 0.16]	39.5
	HGI		< 0.001	[0.03, 0.11]	[− 0.05, 0.07]	[0.01, 0.15]	85.2
	HDL-C/TC		< 0.001	[− 0.10, − 0.03]	[− 0.08, 0.05]	[− 0.15, 0.00]	81.4
DKD	HOMA-IR	286 (39.3)	< 0.001	[0.04, 0.15]	[0.05, 0.27]	[0.12, 0.39]	32.1
	IGI		< 0.001	[− 0.16, − 0.03]	[− 0.38, − 0.18]	[− 0.48, − 0.25]	19.7
	IGI/HOMA-IR		0.08	[− 0.14, 0.00]	[− 0.26, − 0.02]	[− 0.31, − 0.07]	25.4
	Fasting glucose		< 0.001	[0.05, 0.17]	[0.06, 0.27]	[0.15, 0.40]	34.6
	120 min glucose		< 0.001	[0.04, 0.15]	[− 0.01, 0.17]	[0.07, 0.27]	50.1
	HGI		< 0.001	[0.05, 0.20]	[− 0.05, 0.14]	[0.07, 0.26]	66.3
Non-DKD	HDL-C	442 (60.7)	< 0.001	[− 0.13, − 0.03]	[− 0.10, 0.08]	− 0.17, 0.00]	97.1
	HDL-C/TC		< 0.001	[− 0.10, − 0.02]	[− 0.20, − 0.02]	[− 0.25, − 0.08]	36.3

*TE* total effect, *DE* direct effects, *IE* indirect effects, *CI* confidence interval.

Significant IE and TE were found in 3 out of 20 causal mediation models when evaluating renal impairment using UMALB across all observations. 5 indicators (Gutt index, I/G 0 min, 120 min glucose, GHBA1C, and HGI) reflecting IR, β-cell function, and glucose metabolism in the DKD subgroup and no indicator in the non-DKD subgroup showed significant IE and TE (see Table 5 for significant results and Table S6 for full results).

**Table 5:** DE, IE, and TE of MetS components on renal function impairment (UMALB) mediated through serum Mb (Significant).

Group	MetS components	Number of patients (%)	P_IE_	IE [95% CI]	DE [95% CI]	TE [95% CI]	Proportion mediated (%)
All	Fasting C-peptide	728 (100.0)	0.08	[0.00, 0.05]	[0.06, 0.23]	[0.07, 0.27]	13.3
	I/G 0 min		< 0.001	[0.02, 0.08]	[0.01, 0.15]	[0.05, 0.20]	32.9
	HGI		< 0.001	[− 0.07, − 0.01]	[− 0.07, 0.04]	[− 0.11, 0.00]	60.6
DKD	Gutt index	286 (39.3)	0.10	[− 0.01, 0.12]	[− 0.03, 0.17]	[0.01, 0.22]	40.2
	I/G 0 min		< 0.001	[0.02, 0.12]	[− 0.01, 0.22]	[0.04, 0.28]	39.8
	120 min glucose		< 0.001	[− 0.11, − 0.03]	[− 0.25, − 0.08]	[− 0.32, − 0.14]	25.8
	GHBA1C		< 0.001	[− 0.13, − 0.04]	[− 0.15, 0.03]	[− 0.23, − 0.04]	57.2
	HGI		< 0.001	[− 0.11, − 0.03]	[− 0.16, 0.02]	[− 0.23, − 0.04]	49.2

*TE* total effect, *DE* direct effects, *IE* indirect effects, *CI* confidence interval.

Based on the above systematic evaluation, we support that serum Mb is a potential mediator of MetS component-induced renal function impairment. Generally, compared with UMALB, an indicator of renal function impairment, eGFR has more significant IE and TE as well as higher mediated proportion, indicating serum Mb as a mediator mainly participates in the decline of eGFR, that is, the later phase of DKD progression. The results also suggest that lipid metabolism-induced renal function impairment through serum Mb is important in the non-DKD subgroup, whereas IR, β-cell function, and glucose metabolism are important in the DKD subgroup. These may indicate different causes of elevated serum Mb, and therefore different glycemic and/or lipid control strategies should be employed.

## Discussion

### Serum Mb is a potential risk factor for DKD

Current DKD prediction models based on large cohorts of T2DM patients, such as ADVANCE study (2012)^13^, Diabetes Cohort Study (DCS) (2013)^49^, and National Diabetes Register (NDR) (2011)^44^, are generally limited by lack of biomarkers and poor performance. A recently observed relationship between elevated serum Mb and DKD suggests that serum Mb may be a risk factor for DKD^43,45^. However, to our knowledge, no studies have evaluated serum Mb as a risk factor for DKD prediction, nor has serum Mb been used as a risk factor for DKD model construction, partially due to limitations in methods to address collinearity.

We first verified the importance of serum Mb in DKD. To find those features that might be overlooked, the ML algorithms were chosen because they are not constrained by collinearity when processing high-dimensional EHR data. Apart from common DKD-related features, such as renal function features and fasting glucose, we also specially developed ML models including serum Mb. Our DKD models had comparable performance to other models, achieving an AUC of 0.85 in the presence of renal function features and 0.80 after removal of renal function features. Further, in our study, feature importance analysis and SHAP have recognized serum Mb as an important feature. Equally important, RCS found that moderately elevated serum Mb was associated with the risk of DKD, which is consistent with previous findings^17–20^. Therefore, we believe that serum Mb is a potential risk factor for DKD and deserves more attention and further study.

### Serum Mb may mediate MetS component-induced renal function impairment

Current DKD management focuses solely on blood pressure control, glycemic and lipid control, weight loss, and diet^50–52^, in other words, we emphasize the strong association between MetS components and DKD, but more efforts are needed to find appropriate treatment for DKD^53,54^. Recently, elevated Mb have been observed in DM and DKD. In a 200 controlled study of 188 DM patients, serum Mb levels in DM patients were elevated and were much higher in DM patients with metabolic syndrome^16^. In a cross-sectional study of DKD, serum Mb was found to be positively correlated with DKD in a model adjusted for age, gender, cardiovascular risk factors, glycemic control, and blood sugar^45^. In another study, serum Mb was found to be related to CKD, and elevated serum Mb levels were related to advanced stage of CKD^43^. Interestingly, the elevation of serum Mb cannot be explained by Statins uses^55^. The cause of elevated serum Mb may be due to abnormal glucose and lipid metabolism and diabetic muscle tissue damage^17,18^. In diabetic rats, liraglutide and insulin increased carnitine palmitoyl transferase 1 (CPT1) expression, decreased Mb, and improved cardiovascular function^19^. In a skeletal muscle-specific (Cpt1bm-/-) mouse model, a 25% fat diet resulted in increased intramuscular lipids and serum Mb and decreased mouse vitality^20^.

Further, at the molecular level, Mb interacts with glucose to form AGEs^33,56^, which are active contributors to cellular stress and chronic inflammation^34,57^. Also, oxidative modification of Mb induced by stress-related protein carbonyl compounds is an important cause for the development of microvascular and macrovascular complications in diabetes^21–23^.

These cumulative evidence indicate that serum Mb has a mediator role in DKD. Confirming the role of serum Mb in DKD progression may provide a thorough comprehension of DKD pathogenesis and a promising therapeutic target for DKD. However, as far as we known, the causal mediation relationship between serum Mb and MetS components and renal function impairment has not been reported.

In our study, feature importance analysis, SHAP, and RCS revealed that MetS components and elevated serum Mb were closely related to DKD. In causal mediation analysis, significant IE and TE were found using either UMALB or eGFR as indicators of renal function impairment, indicating serum Mb as a mediator in MetS component-induced renal function impairment. Thus, we believe that more studies on the role of serum Mb in DKD, based on multilevel research designs and emerging strategies, will provide insights into the pathogenesis of DKD and reveal new treatment targets for DKD.

### Study Limitations

Our findings should be further verified in cohort studies, as the strength of the evidence we generated is limited by the cross-sectional study design. External validity was difficult to perform in our study because serum Mb was not a commonly used indicator in DKD studies. Further, it is necessary to gain insights into the pathophysiological mechanisms at the cellular and molecular levels to reveal the causal relationship between MetS components, serum Mb, and renal function impairment.

## Supplementary Information

Supplementary Figures.

Supplementary Table S1.

Supplementary Tables S2 to S6.

## Data Availability

The datasets generated during and/or analyzed during the current study are not publicly available usage due to the other ongoing projects but are available from the corresponding author on reasonable request.
