# Supplementary material for: Identifying myoglobin as a mediator of diabetic kidney disease: a machine learning-based cross-sectional study
Source: Sci Rep. 2022 Dec 10;12:21411. doi: 10.1038/s41598-022-25299-8 (PMC9741614; doi:10.1038/s41598-022-25299-8)
Supplement: Supplementary file 1 — Supplementary Figures. [file 41598_2022_25299_MOESM1_ESM.docx]

**Figure S1.** ROC curves and feature importance analysis. (A) ROC curves of RF models based on 51,866 T2DM patients. (B) Feature importance estimated by mean decrease accuracy and mean decreased Gini index.

**Figure S2.** Restricted cubic spline models of the log odds ratios of DKD with serum Mb on a continuous scale. Analyses were adjusted for gender, age, BMI, hyperlipidemia, hypertension, ACCI, hospitalized date and eGFR. (A) Red solid line is multivariable adjusted log odds ratio, with shaded area showing 95% confidence intervals derived from restricted cubic spline regressions with four knots. Dashed red curve shows the distribution density of the population with different levels of serum Mb. (B) Orange and blue solid lines are multivariable adjusted log odds ratio of female and male subgroups respectively, with shaded area showing 95% confidence intervals derived from restricted cubic spline regressions with four knots. Dashed orange and blue curves show the distribution density of the population with different levels of serum Mb in female and male subgroups respectively.

**Figure S3.** The absolute spearman correlation coefficients between 20 MetS components.
