# Supplementary material for: Identifying myoglobin as a mediator of diabetic kidney disease: a machine learning-based cross-sectional study
Source: Sci Rep. 2022 Dec 10;12:21411. doi: 10.1038/s41598-022-25299-8 (PMC9741614; doi:10.1038/s41598-022-25299-8)
Supplement: Supplementary file 2 — Supplementary Table S1. [file 41598_2022_25299_MOESM2_ESM.docx]

| **Table S1. Details of features calculation** | | | | | |
| --- | --- | --- | --- | --- | --- |
| Domain | Abbreviations | Names | Significance | Calculation | Reference |
| General information | BMI | Body-mass index | - | weight in kilo- grams per square meter (kg/m^2^). | [1] |
| Laboratory tests | HGI | Hemoglobin glycation index | Glucose control | observed - predicted HbA1c [%] (predicted HbA1c = 0.009 * fasting plasma glucose [mg/dL] + 6.8) | [2] |
|  | TyG index | Triglyceride-glucose index | Glucose and lipid metabolism | ln [fasting TG (mmol/L) × FPG (mmol/L) × 0.5 × 159.37] | [3] |
|  | TG/HDL ratio | Triacylglycerol to high density lipoprotein cholesterol ratio | Related to IR and progression of DKD | triacylglycerol (mmol/L) / high density lipoprotein cholesterol (mmol/L) | [4] |
|  | HOMA-IR | Homeostatic Model Assessment of Insulin Resistance | IR | fasting plasma insulin (μU/mL) × fasting plasma glucose (mmol/L)/22.5 | [5] |
|  | Gutt index | Gutt index | IR | [75000+(fasting glucose (mmol/L) -120min glucose (mmol/L) )*0.19*weight (kg)]/(120*log[(fasting insulin (μU/mL) +120min insulin (μU/mL) )/2]*[ (fasting glucose (mmol/L) +120min glucose (mmol/L) )/2]) | [6] |
|  | IGI/HOMA-IR | Insulinogenic index to HOMA-IR ratio | Beta-cell function | Insulinogenic index/ HOMA-IR | [7] |
|  | HOMA-BETA | Homeostasis Model of Assessment for beta-cell function | Beta-cell function and IR | fasting plasma insulin (μU/mL) / (fasting plasma glucose (mmol/L)-3.5) | [8] |
|  | IGI | Insulinogenic index | Beta-cell function and IR | [C-peptide 120 min (ng/mL) – C-peptide 0 min (ng/mL)]/[glucose 120 min (mmol/L) – glucose 0 min (mmol/L)] | [9] |
|  | I/G 0min | Fasting Insulin to glucose ratio | Beta-cell function and IR | fasting C-peptide (ng/mL) / fasting glucose (mmol/L) | [10] |
|  | I/G 120min | 120min Insulin to glucose ratio | Beta-cell function and IR | 120min C-peptide (ng/mL) / 120min glucose (mmol/L) | [10] |
|  | eGFR | Estimated glomerular filtration rate | Severity of DKD | eGFR(ml/min/1.73m2) = 186*[serum creatine (mg/dL)]^-1.154^ * (age)^-0.203^ * 0.742(for women) | [11] |
|  | NLR | Neutrophil-to-lymphocyte ratio | Inflammation markers | Neutrophils / lymphocytes | [12] |
|  | PLR | Platelet-to-lymphocyte ratio | Inflammation markers | platelet / lymphocytes | [13] |
| Others | ACCI | Age-adjusted Charlson Comorbidity Index | Classifying prognostic comorbidity | See references for details | [14] |
|  | FRS | Framingham Risk Score | Cardiovascular risk |  | [15] |

1. Parving HH, Lehnert H, Bröchner-Mortensen J, Gomis R, Andersen S, Arner P. The effect of irbesartan on the development of diabetic nephropathy in patients with type 2 diabetes. *N Engl J Med*. 2001;345(12):870-878. doi:10.1056/NEJMoa011489

2. Hempe JM, Liu S, Myers L, McCarter RJ, Buse JB, Fonseca V. The hemoglobin glycation index identifies subpopulations with harms or benefits from intensive treatment in the ACCORD trial. *Diabetes Care*. 2015;38(6):1067-1074. doi:10.2337/dc14-1844

3. Xie Y, Guo R, Li Z, et al. Temporal relationship between body mass index and triglyceride-glucose index and its impact on the incident of hypertension. *Nutr Metab Cardiovasc Dis*. 2019;29(11):1220-1229. doi:10.1016/j.numecd.2019.07.003

4. Block G, Azar KM, Romanelli RJ, et al. Diabetes Prevention and Weight Loss with a Fully Automated Behavioral Intervention by Email, Web, and Mobile Phone: A Randomized Controlled Trial Among Persons with Prediabetes. *J Med Internet Res*. 2015;17(10):e240. doi:10.2196/jmir.4897

5. Mingrone G, Panunzi S, De Gaetano A, et al. Metabolic surgery versus conventional medical therapy in patients with type 2 diabetes: 10-year follow-up of an open-label, single-centre, randomised controlled trial. *Lancet (London, England)*. 2021;397(10271):293-304. doi:10.1016/S0140-6736(20)32649-0

6. Rynders CA, Weltman JY, Malin SK, et al. Comparing Simple Insulin Sensitivity Indices to the Oral Minimal Model Postexercise. *Med Sci Sports Exerc*. 2016;48(1):66-72. doi:10.1249/MSS.0000000000000728

7. Monti LD, Galluccio E, Villa V, Fontana B, Spadoni S, Piatti PM. Decreased diabetes risk over 9 year after 18-month oral L-arginine treatment in middle-aged subjects with impaired glucose tolerance and metabolic syndrome (extension evaluation of L-arginine study). *Eur J Nutr*. 2018;57(8):2805-2817. doi:10.1007/s00394-017-1548-2

8. McKay DL, Eliasziw M, Chen CYO, Blumberg JB. A Pecan-Rich Diet Improves Cardiometabolic Risk Factors in Overweight and Obese Adults: A Randomized Controlled Trial. *Nutrients*. 2018;10(3). doi:10.3390/nu10030339

9. Lemieux P, Weisnagel SJ, Caron AZ, et al. Effects of 6-month vitamin D supplementation on insulin sensitivity and secretion: a randomised, placebo-controlled trial. *Eur J Endocrinol*. 2019;181(3):287-299. doi:10.1530/EJE-19-0156

10. von Post-Skagegård M, Vessby B, Karlström B. Glucose and insulin responses in healthy women after intake of composite meals containing cod-, milk-, and soy protein. *Eur J Clin Nutr*. 2006;60(8):949-954. doi:10.1038/sj.ejcn.1602404

11. Imai E, Horio M, Nitta K, et al. Modification of the Modification of Diet in Renal Disease (MDRD) Study equation for Japan. *Am J kidney Dis Off J Natl Kidney Found*. 2007;50(6):927-937. doi:10.1053/j.ajkd.2007.09.004

12. Ma A, Cheng J, Yang J, Dong M, Liao X, Kang Y. Neutrophil-to-lymphocyte ratio as a predictive biomarker for moderate-severe ARDS in severe COVID-19 patients. *Crit Care*. 2020;24(1):288. doi:10.1186/s13054-020-03007-0

13. Rees CA, Pineros DB, Amour M, et al. The potential of CBC-derived ratios (monocyte-to-lymphocyte, neutrophil-to-lymphocyte, and platelet-to-lymphocyte) to predict or diagnose incident TB infection in Tanzanian adolescents. *BMC Infect Dis*. 2020;20(1):609. doi:10.1186/s12879-020-05331-w

14. Koppie TM, Serio AM, Vickers AJ, et al. Age-adjusted Charlson comorbidity score is associated with treatment decisions and clinical outcomes for patients undergoing radical cystectomy for bladder cancer. *Cancer*. 2008;112(11):2384-2392. doi:10.1002/cncr.23462

15. Nishimura K, Okamura T, Watanabe M, et al. Predicting coronary heart disease using risk factor categories for a Japanese urban population, and comparison with the framingham risk score: the suita study. *J Atheroscler Thromb*. 2014;21(8):784-798. doi:10.5551/jat.19356
