# Supplementary material for: Identifying myoglobin as a mediator of diabetic kidney disease: a machine learning-based cross-sectional study
Source: Sci Rep. 2022 Dec 10;12:21411. doi: 10.1038/s41598-022-25299-8 (PMC9741614; doi:10.1038/s41598-022-25299-8)
Supplement: Supplementary file 3 — Supplementary Tables S2 to S6. [file 41598_2022_25299_MOESM3_ESM.docx]

| **Table S2. Final DKD model hyperparameters** | |
| --- | --- |
| Model | Hyperparameter |
| XGBoost-88 | Step size shrinkage = 0.2, maximum depth of a tree = 8, the max number of iterations = 100 |
| XGBoost-83 | Step size shrinkage = 0.4, maximum depth of a tree = 8, the max number of iterations = 100 |
| RF-88 | The number of trees = 50, mtry = 8 |
| RF-83 | The number of trees = 250, mtry = 12 |

| **Table S3. Demographic and clinical features for bias analysis** | | | | |  |
| --- | --- | --- | --- | --- | --- |
| Variables | Overall (n = 26115) | Excluded patients  (n = 25387) | Eligible patients (n = 728) | p | |
| Male | 15055 (57.7) | 14662 (57.8) | 393 (54.0) | 0.043 | |
| Age | 61.00 [52.00, 69.00] | 61.00 [52.00, 69.00] | 61.00 [52.00, 69.00] | 0.459 | |
| DKD (%) | 3649 (14.0) | 3363 (13.3) | 286 (39.3) | <0.001 | |
| Cardiovascular Disease (%) | 4848 (18.6) | 4728 (18.6) | 120 (16.5) | 0.154 | |
| Hypertension (%) | 14373 (55.1) | 13948 (55.0) | 425 (58.4) | 0.076 | |
| ACCI | 4.00 [3.00, 6.00] | 4.00 [3.00, 6.00] | 6.00 [5.00, 7.00] | <0.001 | |
| FRS | 14.00 [11.00, 16.00] | 14.00 [11.00, 16.00] | 14.00 [12.00, 16.00] | 0.246 | |
| UMALB (mg/L) | 20.00 [9.70, 71.00] | 20.00 [9.60, 65.32] | 25.95 [10.88, 188.42] | <0.001 | |
| serum Mb (ng/mL) | 54.70 [39.60, 77.90] | 54.60 [39.50, 77.00] | 56.85 [41.20, 88.10] | 0.004 | |
| eGFR (ml/min/1.73m2) | 90.90 [67.70, 112.74] | 91.33 [68.14, 113.15] | 87.93 [65.11, 110.87] | 0.003 | |
| Data are presented as median [interquartile range] for continuous variables or number (%) for categorical variables. P-values for differences between groups were based on the Wilcoxon test for continuous variables and the chi-square test for categorical variables. Details of features calculation are shown in Table S1. Abbreviations: ACCI: age-adjusted Charlson Comorbidity Index; FRS: Framingham Risk Score; UMALB: urinary albumin excretion; eGFR: estimated glomerular filtration rate; Mb: myoglobin | | | | | |

| **Table S4. Spearman correlation coefficient between MetS components, serum Mb and renal function indicators** | | | | |
| --- | --- | --- | --- | --- |
| variables | variables | r_s_ | t value | P-value |
| eGFR | Mb | -0.577 | -19.049 | <0.001 |
|  | Fasting C-peptide | -0.216 | -5.954 | <0.001 |
|  | 120 min C-peptide | -0.099 | -2.679 | 0.008 |
|  | HOMA-IR | -0.049 | -1.324 | 0.186 |
|  | Gutt index | 0.037 | 0.992 | 0.321 |
|  | TyG index | 0.049 | 1.328 | 0.184 |
|  | I/G 0 min | -0.276 | -7.724 | <0.001 |
|  | I/G 120 min | -0.137 | -3.737 | <0.001 |
|  | IGI | -0.016 | -0.422 | 0.673 |
|  | IGI/HOMA-IR | 0.012 | 0.333 | 0.739 |
|  | HOMA-BETA | -0.139 | -3.775 | <0.001 |
|  | Fasting glucose | 0.114 | 3.095 | 0.002 |
|  | 120 min glucose | 0.052 | 1.415 | 0.157 |
|  | GHBA1C | 0.213 | 5.880 | <0.001 |
|  | HGI | 0.217 | 6.004 | <0.001 |
|  | TC | 0.053 | 1.433 | 0.152 |
|  | TG | -0.008 | -0.214 | 0.831 |
|  | LDL-C | 0.090 | 2.428 | 0.015 |
|  | HDL-C | -0.019 | -0.521 | 0.602 |
|  | HDL-C/TC | -0.075 | -2.017 | 0.044 |
|  | TG/HDL ratio | -0.027 | -0.717 | 0.473 |
| UMALB | Mb | 0.403 | 11.856 | <0.001 |
|  | Fasting C-peptide | 0.161 | 4.400 | <0.001 |
|  | 120 min C-peptide | -0.022 | -0.601 | 0.548 |
|  | HOMA-IR | 0.010 | 0.259 | 0.795 |
|  | Gutt index | 0.060 | 1.614 | 0.107 |
|  | TyG index | 0.077 | 2.083 | 0.038 |
|  | I/G 0 min | 0.117 | 3.182 | 0.002 |
|  | I/G 120 min | -0.012 | -0.330 | 0.741 |
|  | IGI | -0.012 | -0.324 | 0.746 |
|  | IGI/HOMA-IR | -0.025 | -0.667 | 0.505 |
|  | HOMA-BETA | -0.014 | -0.380 | 0.704 |
|  | Fasting glucose | 0.025 | 0.685 | 0.493 |
|  | 120 min glucose | -0.062 | -1.681 | 0.093 |
|  | GHBA1C | -0.034 | -0.912 | 0.362 |
|  | HGI | -0.049 | -1.326 | 0.185 |
|  | TC | 0.033 | 0.891 | 0.373 |
|  | TG | 0.032 | 0.867 | 0.386 |
|  | LDL-C | -0.042 | -1.142 | 0.254 |
|  | HDL-C | -0.060 | -1.611 | 0.108 |
|  | HDL-C/TC | -0.081 | -2.198 | 0.028 |
|  | TG/HDL ratio | -0.030 | -0.806 | 0.421 |
| Mb | Fasting C-peptide | 0.153 | 4.162 | <0.001 |
|  | 120 min C-peptide | 0.069 | 1.858 | 0.064 |
|  | HOMA-IR | 0.041 | 1.116 | 0.265 |
|  | Gutt index | 0.004 | 0.095 | 0.924 |
|  | TyG index | -0.045 | -1.219 | 0.223 |
|  | I/G 0 min | 0.199 | 5.459 | <0.001 |
|  | I/G 120 min | 0.129 | 3.512 | <0.001 |
|  | IGI | 0.024 | 0.656 | 0.512 |
|  | IGI/HOMA-IR | 0.003 | 0.086 | 0.931 |
|  | HOMA-BETA | 0.118 | 3.193 | 0.001 |
|  | Fasting glucose | -0.067 | -1.801 | 0.072 |
|  | 120 min glucose | -0.064 | -1.716 | 0.087 |
|  | GHBA1C | -0.163 | -4.459 | <0.001 |
|  | HGI | -0.169 | -4.607 | <0.001 |
|  | TC | 0.051 | 1.379 | 0.168 |
|  | TG | 0.035 | 0.956 | 0.340 |
|  | LDL-C | -0.001 | -0.019 | 0.985 |
|  | HDL-C | 0.110 | 2.978 | 0.003 |
|  | HDL-C/TC | 0.057 | 1.550 | 0.122 |
|  | TG/HDL ratio | 0.036 | 0.972 | 0.331 |

Spearman correlation coefficients were adjusted by 7 variables: gender, age, BMI, hyperlipidemia, hypertension, ACCI and hospitalization date.

| **Table S5. DE, IE, and TE of MetS components on renal function impairment (eGFR) mediated through serum Mb** | | | | | | | |
| --- | --- | --- | --- | --- | --- | --- | --- |
| Group | MetS components | Number of patients (%) | P_IE_ | IE [95%CI] | DE [95%CI] | TE [95%CI] | Proportion Medited |
| ALL | Fasting C-peptide | 728 (100.0) | 0.02 | [-0.07, -0.01] | [-0.30, -0.16] | [-0.35, -0.20] | 0.135 |
|  | 120min C-peptide |  | 0.86 | [-0.04, 0.03] | [-0.17, -0.04] | [-0.19, -0.04] | 0.034 |
|  | HOMA-IR |  | <0.001 | [0.02, 0.08] | [0.09, 0.22] | [0.15, 0.28] | 0.221 |
|  | Gutt index |  | 0.62 | [-0.05, 0.03] | [-0.07, 0.07] | [-0.08, 0.06] | 0.590 |
|  | TyG index |  | 0.48 | [-0.08, 0.09] | [-0.15, -0.02] | [-0.19, 0.00] | 0.211 |
|  | I/G 0min |  | 0.24 | [-0.07, 0.03] | [0.02, 0.13] | [-0.03, 0.12] | -0.591 |
|  | I/G 120min |  | 0.16 | [-0.04, 0.00] | [-0.03, 0.06] | [-0.04, 0.05] | -1.018 |
|  | IGI |  | <0.001 | [-0.10, -0.03] | [-0.32, -0.19] | [-0.39, -0.26] | 0.176 |
|  | IGI/HOMA-IR |  | 0.02 | [-0.07, 0.00] | [-0.17, -0.04] | [-0.21, -0.08] | 0.206 |
|  | HOMA-BETA |  | 0.62 | [-0.34, 0.16] | [-0.19, 0.53] | [-0.15, 0.46] | 0.137 |
|  | Fasting glucose |  | <0.001 | [0.02, 0.09] | [0.10, 0.21] | [0.15, 0.28] | 0.239 |
|  | 120min glucose |  | 0.04 | [0.00, 0.07] | [0.00, 0.11] | [0.03, 0.16] | 0.395 |
|  | GHBA1C |  | 0.04 | [0.01, 0.08] | [-0.06, 0.05] | [-0.02, 0.11] | 0.888 |
|  | HGI |  | <0.001 | [0.03, 0.11] | [-0.05, 0.07] | [0.01, 0.15] | 0.852 |
|  | TC |  | 0.02 | [-0.08, -0.01] | [0.04, 0.17] | [-0.02, 0.14] | -0.686 |
|  | TG |  | 0.80 | [-0.04, 0.04] | [-0.03, 0.10] | [-0.04, 0.08] | 0.096 |
|  | LDL-C |  | 0.44 | [-0.08, 0.03] | [0.06, 0.18] | [0.02, 0.18] | -0.160 |
|  | HDL-C |  | <0.001 | [-0.15, -0.07] | [0.00, 0.15] | [-0.12, 0.04] | 2.920 |
|  | HDL-C/TC |  | <0.001 | [-0.10, -0.03] | [-0.08, 0.05] | [-0.15, 0.00] | 0.814 |
|  | TG/HDL ratio |  | 0.96 | [-0.06, 0.07] | [-0.06, 0.10] | [-0.06, 0.09] | -0.307 |
| DKD | Fasting C-peptide | 286 (39.3) | 0.16 | [-0.11, 0.01] | [-0.34, -0.16] | [-0.39, -0.17] | 0.122 |
|  | 120min C-peptide |  | 0.84 | [-0.06, 0.03] | [-0.25, -0.03] | [-0.27, -0.05] | 0.013 |
|  | HOMA-IR |  | <0.001 | [0.04, 0.15] | [0.05, 0.27] | [0.12, 0.39] | 0.321 |
|  | Gutt index |  | 0.68 | [-0.02, 0.06] | [-0.03, 0.13] | [-0.03, 0.16] | 0.129 |
|  | TyG index |  | 0.62 | [-0.18, 0.10] | [-0.16, 0.03] | [-0.24, 0.00] | 0.334 |
|  | I/G 0min |  | 0.50 | [-0.09, 0.04] | [0.05, 0.25] | [0.00, 0.29] | -0.225 |
|  | I/G 120min |  | 0.04 | [-0.13, 0.00] | [-0.10, 0.13] | [-0.17, 0.05] | 1.855 |
|  | IGI |  | <0.001 | [-0.16, -0.03] | [-0.38, -0.18] | [-0.48, -0.25] | 0.197 |
|  | IGI/HOMA-IR |  | 0.08 | [-0.14, 0.00] | [-0.26, -0.02] | [-0.31, -0.07] | 0.254 |
|  | HOMA-BETA |  | 0.10 | [-0.17, 0.01] | [0.01, 0.28] | [-0.09, 0.24] | -1.441 |
|  | Fasting glucose |  | <0.001 | [0.05, 0.17] | [0.06, 0.27] | [0.15, 0.40] | 0.346 |
|  | 120min glucose |  | <0.001 | [0.04, 0.15] | [-0.01, 0.17] | [0.07, 0.27] | 0.501 |
|  | GHBA1C |  | 0.02 | [0.02, 0.16] | [-0.12, 0.06] | [-0.06, 0.18] | 1.665 |
|  | HGI |  | <0.001 | [0.05, 0.20] | [-0.05, 0.14] | [0.07, 0.26] | 0.663 |
|  | TC |  | <0.001 | [-0.15, -0.02] | [-0.00, 0.22] | [-0.08, 0.12] | -10.357 |
|  | TG |  | 0.82 | [-0.13, 0.07] | [-0.05, 0.21] | [-0.05, 0.16] | -0.365 |
|  | LDL-C |  | 0.40 | [-0.14, 0.04] | [-0.05, 0.18] | [-0.07, 0.15] | -1.795 |
|  | HDL-C |  | <0.001 | [-0.18, -0.05] | [-0.01, 0.21] | [-0.15, 0.14] | 4.409 |
|  | HDL-C/TC |  | 0.08 | [-0.14, 0.01] | [-0.09, 0.12] | [-0.17, 0.09] | 1.326 |
|  | TG/HDL ratio |  | 0.74 | [-0.17, 0.10] | [-0.09, 0.20] | [-0.07, 0.12] | -2.373 |
| Non-DKD | Fasting C-peptide | 442 (60.7) | 0.68 | [-0.05, 0.03] | [-0.28, -0.09] | [-0.30, -0.11] | 0.012 |
|  | 120min C-peptide |  | 0.76 | [-0.04, 0.03] | [-0.20, -0.01] | [-0.20, -0.01] | 0.006 |
|  | HOMA-IR |  | 0.28 | [-0.02, 0.06] | [0.11, 0.25] | [0.11, 0.29] | 0.072 |
|  | Gutt index |  | 0.40 | [-0.05, 0.03] | [-0.18, 0.04] | [-0.20, 0.01] | 0.191 |
|  | TyG index |  | 0.34 | [-0.04, 0.01] | [-0.23, -0.04] | [-0.25, -0.04] | 0.069 |
|  | I/G 0min |  | 0.52 | [-0.08, 0.04] | [-0.07, 0.07] | [-0.10, 0.08] | 9.774 |
|  | I/G 120min |  | 0.48 | [-0.01, 0.03] | [-0.04, 0.09] | [-0.03, 0.10] | 0.142 |
|  | IGI |  | 0.52 | [-0.05, 0.02] | [-0.31, -0.14] | [-0.32, -0.15] | 0.032 |
|  | IGI/HOMA-IR |  | 0.28 | [-0.05, 0.01] | [-0.23, -0.05] | [-0.25, -0.07] | 0.094 |
|  | HOMA-BETA |  | 0.72 | [-0.21, 0.16] | [-0.21, 0.46] | [-0.24, 0.40] | 0.000 |
|  | Fasting glucose |  | 0.32 | [-0.02, 0.06] | [0.10, 0.25] | [0.09, 0.27] | 0.070 |
|  | 120min glucose |  | 0.72 | [-0.03, 0.03] | [-0.04, 0.12] | [-0.05, 0.12] | 0.038 |
|  | GHBA1C |  | 0.58 | [-0.04, 0.03] | [-0.07, 0.11] | [-0.08, 0.12] | 0.195 |
|  | HGI |  | 0.06 | [0.00, 0.07] | [-0.07, 0.09] | [-0.04, 0.13] | 0.649 |
|  | TC |  | 0.66 | [-0.04, 0.04] | [0.05, 0.22] | [0.03, 0.22] | -0.079 |
|  | TG |  | 0.14 | [-0.01, 0.04] | [-0.06, 0.05] | [-0.06, 0.07] | 1.966 |
|  | LDL-C |  | 0.7 | [-0.05, 0.04] | [0.08, 0.26] | [0.04, 0.26] | -0.070 |
|  | HDL-C |  | <0.001 | [-0.13, -0.03] | [-0.10, 0.08] | [-0.17, 0.00] | 0.971 |
|  | HDL-C/TC |  | <0.001 | [-0.10, -0.02] | [-0.20, -0.02] | [-0.25, -0.08] | 0.363 |
|  | TG/HDL ratio |  | 0.10 | [0.00. 0.04] | [-0.08, 0.06] | [-0.07, 0.09] | 13.200 |

Abbreviations: TE: total effect; DE: direct effects; IE: indirect effects; CI: confidence interval.

| **Table S6. DE, IE, and TE of MetS components on renal function impairment (UMALB) mediated through serum Mb** | | | | | | | |
| --- | --- | --- | --- | --- | --- | --- | --- |
| Group | MetS components | Number of patients (%) | P_IE_ | IE [95%CI] | DE [95%CI] | TE [95%CI] | Proportion Medited (%) |
| ALL | Fasting C-peptide | 728 (100.0) | 0.08 | [0.00, 0.05] | [0.06, 0.23] | [0.07, 0.27] | 0.133 |
|  | 120 min C-peptide |  | 0.18 | [-0.04, 0.01] | [-0.09, 0.04] | [-0.11, 0.02] | 0.333 |
|  | HOMA-IR |  | 0.94 | [-0.02, 0.03] | [-0.08, 0.01] | [-0.09, 0.02] | -0.069 |
|  | Gutt index |  | 0.62 | [-0.12, 0.22] | [-0.22, 0.52] | [-0.22, 0.57] | -0.050 |
|  | TyG index |  | <0.001 | [-0.09, -0.03] | [0.03, 0.15] | [-0.03, 0.10] | -1.390 |
|  | I/G 0 min |  | <0.001 | [0.02, 0.08] | [0.01, 0.15] | [0.05, 0.20] | 0.329 |
|  | I/G 120 min |  | 0.02 | [0.00, 0.06] | [-0.10, -0.01] | [-0.09, 0.02] | -0.784 |
|  | IGI |  | 0.24 | [-0.02, 0.06] | [-0.13, 0.03] | [-0.12, 0.05] | -0.628 |
|  | IGI/HOMA-IR |  | 0.16 | [-0.00, 0.03] | [-0.02, 0.14] | [-0.01, 0.16] | 0.169 |
|  | HOMA-BETA |  | 0.34 | [-0.05, 0.07] | [-0.06, 0.08] | [-0.08, 0.14] | 0.538 |
|  | Fasting glucose |  | <0.001 | [-0.07, -0.01] | [-0.06, 0.09] | [-0.10, 0.05] | 1.389 |
|  | 120 min glucose |  | <0.001 | [-0.06, -0.01] | [-0.12, 0.00] | [-0.17, -0.04] | 0.343 |
|  | GHBA1C |  | <0.001 | [-0.07, -0.01] | [-0.06, 0.06] | [-0.11, 0.01] | 0.807 |
|  | HGI |  | <0.001 | [-0.07, -0.01] | [-0.07, 0.04] | [-0.11, 0.00] | 0.606 |
|  | TC |  | 0.38 | [-0.03, 0.02] | [0.03, 0.16] | [0.02, 0.17] | -0.126 |
|  | TG |  | <0.001 | [-0.06, -0.02] | [0.02, 0.14] | [-0.02, 0.12] | -0.805 |
|  | LDL-C |  | 0.36 | [-0.04, 0.02] | [-0.02, 0.10] | [-0.04, 0.09] | -0.587 |
|  | HDL-C |  | <0.001 | [0.02, 0.09] | [-0.10, 0.01] | [-0.05, 0.07] | 17.222 |
|  | HDL-C/TC |  | <0.001 | [0.04, 0.1] | [-0.20, -0.08] | [-0.14, -0.01] | -0.962 |
|  | TG/HDL ratio |  | 0.02 | [-0.05, 0.00] | [0.00, 0.12] | [-0.02, 0.11] | -0.557 |
| DKD | Fasting C-peptide | 286 (39.3) | 0.36 | [-0.02, 0.08] | [0.02, 0.28] | [0.04, 0.31] | 0.106 |
|  | 120 min C-peptide |  | 0.44 | [-0.05, 0.03] | [-0.17, 0.06] | [-0.19, 0.06] | 0.242 |
|  | HOMA-IR |  | 0.50 | [-0.05, 0.02] | [-0.13, 0.05] | [-0.15, 0.02] | 0.149 |
|  | Gutt index |  | 0.10 | [-0.01, 0.12] | [-0.03, 0.17] | [0.01, 0.22] | 0.402 |
|  | TyG index |  | <0.001 | [-0.16, -0.06] | [-0.02, 0.19] | [-0.11, 0.10] | 7.874 |
|  | I/G 0 min |  | <0.001 | [0.02, 0.12] | [-0.01, 0.22] | [0.04, 0.28] | 0.398 |
|  | I/G 120 min |  | 0.08 | [-0.00, 0.12] | [-0.16, 0.05] | [-0.12, 0.09] | -1.176 |
|  | IGI |  | 0.50 | [-0.03, 0.07] | [-0.21, 0.02] | [-0.20, 0.05] | -0.248 |
|  | IGI/HOMA-IR |  | 0.04 | [0.00, 0.10] | [-0.11, 0.16] | [-0.04, 0.20] | 0.306 |
|  | HOMA-BETA |  | 0.58 | [-0.06, 0.13] | [-0.06, 0.17] | [-0.06, 0.19] | 0.388 |
|  | Fasting glucose |  | <0.001 | [-0.12, -0.04] | [-0.11, 0.07] | [-0.20, 0.01] | 0.777 |
|  | 120 min glucose |  | <0.001 | [-0.11, -0.03] | [-0.25, -0.08] | [-0.32, -0.14] | 0.258 |
|  | GHBA1C |  | <0.001 | [-0.13, -0.04] | [-0.15, 0.03] | [-0.23, -0.04] | 0.572 |
|  | HGI |  | <0.001 | [-0.11, -0.03] | [-0.16, 0.02] | [-0.23, -0.04] | 0.492 |
|  | TC |  | 0.94 | [-0.04, 0.05] | [0.07, 0.29] | [0.06, 0.32] | 0.036 |
|  | TG |  | <0.001 | [-0.09, -0.02] | [0.02, 0.25] | [-0.04, 0.20] | -0.871 |
|  | LDL-C |  | 0.88 | [-0.05, 0.06] | [-0.04, 0.22] | [-0.07, 0.24] | -0.022 |
|  | HDL-C |  | <0.001 | [0.02, 0.14] | [-0.11, 0.08] | [-0.05, 0.18] | 1.059 |
|  | HDL-C/TC |  | <0.001 | [0.03, 0.16] | [-0.26, -0.06] | [-0.18, 0.05] | -1.904 |
|  | TG/HDL ratio |  | 0.04 | [-0.09, 0.00] | [-0.01, 0.15] | [-0.08, 0.15] | -1.208 |
| Non-DKD | Fasting C-peptide | 442 (60.7) | 0.98 | [-0.01, 0.02] | [-0.06, 0.12] | [-0.06, 0.13] | -0.025 |
|  | 120 min C-peptide |  | 0.48 | [-0.02, 0.01] | [-0.10, 0.06] | [-0.10, 0.05] | 0.147 |
|  | HOMA-IR |  | 0.68 | [-0.01, 0.02] | [-0.11, -0.01] | [-0.11, 0.00] | -0.053 |
|  | Gutt index |  | 0.78 | [-0.06, 0.08] | [-0.22, 0.47] | [-0.21, 0.48] | 0.000 |
|  | TyG index |  | 0.18 | [-0.04, 0.00] | [0.00, 0.15] | [-0.02, 0.14] | -0.151 |
|  | I/G 0 min |  | 0.58 | [-0.01, 0.03] | [-0.09, 0.10] | [-0.09, 0.11] | 0.371 |
|  | I/G 120 min |  | 0.32 | [-0.01, 0.03] | [-0.14, 0.00] | [-0.14, 0.01] | -0.090 |
|  | IGI |  | 0.54 | [-0.02, 0.05] | [-0.12, 0.06] | [-0.10, 0.06] | -0.372 |
|  | IGI/HOMA-IR |  | 0.46 | [-0.01, 0.00] | [-0.07, 0.10] | [-0.08, 0.10] | 0.613 |
|  | HOMA-BETA |  | 0.16 | [0.00, 0.02] | [-0.15, 0.02] | [-0.15, 0.03] | -0.252 |
|  | Fasting glucose |  | 0.84 | [-0.01, 0.01] | [-0.02, 0.16] | [-0.02, 0.16] | -0.024 |
|  | 120 min glucose |  | 0.42 | [-0.02, 0.01] | [-0.08, 0.19] | [-0.07, 0.19] | -0.075 |
|  | GHBA1C |  | 0.36 | [-0.03, 0.00] | [-0.01, 0.19] | [-0.02, 0.18] | -0.065 |
|  | HGI |  | 0.32 | [-0.03, 0.00] | [-0.03, 0.18] | [-0.03, 0.17] | -0.074 |
|  | TC |  | 0.08 | [-0.03, 0.00] | [-0.08, 0.09] | [-0.10, 0.08] | 1.287 |
|  | TG |  | 0.08 | [-0.02, 0.00] | [-0.01, 0.13] | [-0.03, 0.12] | -0.209 |
|  | LDL-C |  | 0.12 | [-0.03, 0.00] | [-0.10, 0.07] | [-0.12, 0.07] | 0.316 |
|  | HDL-C |  | 0.34 | [0.00, 0.03] | [-0.15, -0.02] | [-0.14, -0.01] | -0.120 |
|  | HDL-C/TC |  | 0.02 | [0.00, 0.05] | [-0.19, -0.03] | [-0.17, 0.00] | -0.236 |
|  | TG/HDL ratio |  | 0.52 | [-0.01, 0.01] | [-0.01, 0.19] | [-0.02, 0.20] | -0.024 |

Abbreviations: TE: total effect; DE: direct effects; IE: indirect effects; CI: confidence interval.
